# Global health opportunities within pediatric subspecialty fellowship training programs: surveying the virtual landscape

**DOI:** 10.1186/1472-6920-13-88

**Published:** 2013-06-20

**Authors:** Cinnamon A Dixon, Jonathan Castillo, Heidi Castillo, Katherine A Hom, Charles Schubert

**Affiliations:** 1 Division of Emergency Medicine, Department of Pediatrics, Cincinnati Children's Hospital Medical Center, University of Cincinnati, Cincinnati, USA; 2 Center for Global Health, Department of Pediatrics, Cincinnati Children’s Hospital Medical Center, University of Cincinnati, Cincinnati, USA; 3 Meyer Center for Developmental Pediatrics, Baylor College of Medicine, Texas Children’s Hospital, Houston, USA; 4 School of Medicine, University of California – Irvine, Irvine, USA

**Keywords:** Global health, Pediatrics, Graduate medical education, Subspecialty, Fellowship training

## Abstract

**Background:**

There is growing interest in global health among medical trainees. Medical schools and residencies are responding to this trend by offering global health opportunities within their programs. Among United States (US) graduating pediatric residents, 40% choose to subspecialize after residency training. There is limited data, however, regarding global health opportunities within traditional post-residency, subspecialty fellowship training programs. The objectives of this study were to explore the availability and type of global health opportunities within Accreditation Council for Graduate Medical Education (ACGME)-accredited pediatric subspecialty fellowship training programs, as noted by their online report, and to document change in these opportunities over time.

**Methods:**

The authors performed a systematic online review of ACGME-accredited fellowship training programs within a convenience sample of six US pediatric subspecialties. Utilizing two data sources, the American Medical Association-Fellowship and Residency Electronic Interactive Database Access (AMA-FREIDA) and individual program websites, all programs were coded for global health opportunities and opportunity types were stratified into predefined categories. Comparisons were made between 2008 and 2011 using Fisher exact test. All analyses were conducted using SAS Software v. 9.3 (SAS Institute Inc., Cary, NC).

**Results:**

Of the 355 and 360 programs reviewed in 2008 and 2011 respectively, there was an increase in total number of programs listing global health opportunities on AMA-FREIDA (16% to 23%, p=0.02) and on individual program websites (8% to 16%, p=0.004). Nearly all subspecialties had an increased percentage of programs offering global health opportunities on both data sources; although only critical care experienced a significant increase (p=0.04, AMA-FREIDA). The types of opportunities differed across all subspecialties.

**Conclusions:**

Global health opportunities among ACGME-accredited pediatric subspecialty fellowship programs are limited, but increasing as noted by their online report. The availability and types of these opportunities differ by pediatric subspecialty.

## Background

Every year, 6.9 million children under the age of five, die worldwide [1]. The majority of these child deaths occur in developing countries from preventable and/or treatable causes. The United Nation’s Millennium Development Goal 4 is aimed at decreasing child mortality; yet there exists an incredible shortage of health care workers to assist in this effort [2]. In 2007, the extent of the issue was estimated as a deficiency of over 4.2 million health care workers with the shortage most amplified in low and middle income countries. For example, the African region, which represents nearly 50% of global child mortality [3], is served by only 2.8% of the world’s total health workforce [4].

There is a growing interest and desire to help fill this global health workforce gap. In the United States (US), over 30% of medical students have participated in a global health experience by the time they graduate [5]; and one third of graduating pediatric residents plan to work or volunteer in a developing country after residency [6]. Furthermore, many pediatric residents find global health opportunities an essential factor in deciding which residency program to choose [6].

Medical schools and residency programs have begun responding to this trend by incorporating global health training into their programs by developing structured curriculums, increasing the availability of global health rotations and research experiences, and in some cases creating dedicated global health tracks [6-11]. Within traditional pediatric subspecialty fellowship training programs, however, the extent of global health opportunities remains unknown, despite that 40% of annual US graduating pediatric residents choose to subspecialize [12].

With this background in mind, the purpose of this study was to explore the extent of global health opportunities available to potential pediatric fellow applicants interested in both global health and traditional subspecialty training. Our objectives were to describe the availability and type of global health opportunities within a sample of Accreditation Council for Graduate Medical Education (ACGME)-accredited pediatric fellowship training programs, as noted by their online report, and to document change in these opportunities over the typical pediatric subspecialty fellowship length of three years. Given that today’s applicants utilize web-based directories or sites extensively throughout application decision-making processes [13], we sought to assess these opportunities utilizing the two primary online data sources available to potential subspecialty fellow applicants.

## Methods

This descriptive comparison study utilized a systematic online review of ACGME-accredited subspecialty fellowship training programs within a convenience sample of US pediatric subspecialties: adolescent medicine (AM), critical care (CC), emergency medicine (EM), hematology/oncology (H/O), infectious disease (ID), and neonatology-perinatology (NEO). The specific six pediatric subspecialties were selected for their known potential contributions to global health, and because combined they account for at least 50% of pediatric subspecialty fellowship training programs and 60% of all pediatric subspecialty fellows [12]. Programs were identified by the online ACGME-accredited program specialty database and compared between years 2008 and 2011.

Data collection for all subspecialty training programs utilized both the American Medical Association-Fellowship and Residency Electronic Interactive Database Access (AMA-FREIDA) and individual subspecialty training program websites (see Additional file 1: Table S1). On AMA-FREIDA, individual programs list information about the fellowship, including the option to post “yes” or “no” to a specific category of “international experience”. All programs within the six subspecialties were reviewed and coded for the availability of “international experience” on this site. Individual subspecialty training program websites were identified and accessed either via AMA-FREIDA, American Medical Colleges Electronic Residency Application Service or by using Google search engine. Once the program website was found, the entire website was queried for keywords “global” or “international”, and further coded into predetermined categories of opportunity.

Data were described using means and standard deviations or frequencies and percentages. Comparison of categorical variables between groups used Fisher exact test. All analyses were conducted using SAS Software v. 9.3 (SAS Institute Inc., Cary, NC).

## Results

A total of 355 and 360 programs within the six subspecialties were reviewed in the two respective years via both data sources. The number of individual programs in each subspecialty ranged between 25–97 in 2008 and 26–96 in 2011. Three of the six subspecialties had an increase in the total number of programs (see Additional file 2: Table S2).

### AMA-FREIDA

Within the AMA-FREIDA database, the total percentage of programs listing “international experiences” was 16% (57/355) in 2008 and 23% (82/360) in 2011 (p=0.02). An explicit “no” to the category of available “international experience” was reported in 35% (125/355) and 33% (119/360) of programs in the respective years. The remaining 49% (173/355) of programs in 2008 and 44% (159/360) of programs in 2011 had no information available regarding “international experience”. All subspecialties had an increase in percentage of programs posting “international experience” over three years: 8% (AM), 16% (CC), 5% (EM), 7% (H/O), 2% (ID) and 4% (NEO) (see Additional file 2: Table S2).

### Program websites

On individual program websites in years 2008 and 2011, 8% (30/355) and 16% (56/360) of the programs posted “global” or “international” opportunities, respectively (p=0.004). Among specific subspecialties, nearly all had increased percentage of programs listing global health opportunities from 2008 to 2011: 0% (AM), 8% (CC), 7% (EM), 9% (H/O), 9% (ID) and 7% (NEO) (see Additional file 2: Table S2).

The total number of global health opportunity types (elective + research + track + “other”) posted on individual program websites across all specialties increased from 36 in 2008 to 67 in 2011 (see Additional file 3: Table S3). Research and “other” represented the largest percentage of offerings at 30% and 39% in 2008, and 42% and 28% in 2011. The infectious disease subspecialty (ID) accounted for 47% and 38% of opportunity types in 2008 and 2011, respectively. Emergency medicine (EM) was the only subspecialty to offer track opportunities both years.

## Discussion

Despite a growing interest to help fill the global health workforce gap, residents who desire ACGME-accredited subspecialty training have limited resources summarizing the extent of global health opportunities within potential US subspecialty training programs. This study reveals the reported availability and growth of such global health opportunities among a sample of pediatric subspecialty training fellowships, and to the best of our knowledge, is the first of its kind to evaluate these opportunities types among subspecialty training programs over time.

Comparing the two years analyzed, our results show that although there is limited report of global health opportunities among ACGME-accredited pediatric subspecialty fellowship training programs, there has been significant increase in the total number of fellowship programs reporting such opportunities (7% on AMA-FREIDA; 8% on individual program websites). This increase in program offerings was seen across all six subspecialties on AMA-FREIDA (range 2-16%), and for all but one subspecialty adolescent medicine (AM) on individual program websites (range 0-9%). Additionally, the total number of opportunity types within global health (elective + research + other + track) listed among all programs increased nearly two-fold within the three year period. The largest of these increases were seen in research and elective opportunities (61% and 50%, respectively); other opportunity types and official global health tracks had minimal to no reported growth. Certain subspecialties, such as critical care (CC), had more significant increases in the overall number of opportunities in the three year span (p=0.04, AMA-FREIDA) while subspecialties such as infectious disease (ID) and emergency medicine (EM) had more expansive opportunity types both years.

These findings of limited but increasing global health opportunities among ACGME-accredited subspecialty fellowship training programs reveal an important trend that has not previously been described. Pediatric educators are seeing increased critical need for the development of global health opportunities both within residency programs and subspecialty fellowships. This trend is not surprising considering one-third of pediatric residents desire to have global health as part of their careers [5], and one would expect at least a portion of such residents are simultaneously interested in and pursuing subspecialty fellowship training. Whereas residency programs have begun to develop global health educational programs and there is growing consensus on the development of such programs [14,15], subspecialty programs may also be experiencing formative changes based on trainee needs and/or desires, with much more nascent programmatic development. This lack of consensus on subspecialty-based global health fellowship education, along with other pertinent infrastructure/resource items such as cost, leadership, and institutional support may contribute to the disparity in global health offerings across programs and subspecialties. Additionally, though we anticipate that global health opportunities provide benefit beyond personal/educational enrichment of fellows as these subspecialty trained pediatricians can later contribute both general pediatrics and subspecialty expertise abroad, no data on the outcomes of increasing global exposure in subspecialty fellowship exist. This lack of outcome data may further deter programs from creating these opportunities. Research is thus warranted to formally assess the continued growth and development of this field, to evaluate whether fellows with global health opportunities acquire knowledge and skills that are later used in their careers, to explore the capacity in which subspecialty pediatricians trained in global health are contributing their expertise, and lastly, to determine whether global health experiences in fellowship translate to improved global child health.

In addition to increased global health opportunities, our study also highlights an important potential information gap. We found that there was limited information on both data sources regarding the availability of global health opportunities among traditional subspecialty fellowship training programs. On AMA-FREIDA, the national medical site for resident and fellow applicants to access information regarding ACGME-accredited training programs, nearly half of all programs reviewed posted no information about “international experience” both years. Moreover, when comparing the two sources, individual program websites posted significantly less (50% in 2008; 30% in 2011) global health opportunities than AMA-FREIDA. More research is needed to describe and evaluate the extent of possible programmatic mismatch between these data sources. However, given that nearly 70% of trainee applicants utilize web-based sites throughout application decision-making process [13] we would expect accurate identification of global health opportunities on these venues to be beneficial to residents identifying and selecting potential programs, and to programs identifying and recruiting prospective applicants.

The results of our study should be interpreted within the context of its limitations. First, subspecialty fellowship training programs are dynamic and ever changing, and the review of program offerings via the national online database and individual program websites may not reflect actual program opportunities at the time of the online description. In addition, a significant portion of programs had no information posted regarding “international experience” on AMA-FREIDA and/or no mention of global health opportunities on individual program websites, which could limit the interpretation of number of programs offering global health opportunities. Our study, however, aimed at identifying online report of these opportunities on these two data sources, as they are utilized extensively by potential applicants. We would anticipate that keeping the online report of such programmatic opportunities up-to-date could be advantageous to both programs and applicants. Second, our assessment occurred at two discreet points in time, therefore information reported in this manuscript might not represent the current state of opportunities listed on these data sources, and descriptions might have changed since our data collection. We would encourage additional evaluation of programs to include expanding time periods, data sources and subspecialty fellowship training programs. Lastly, our sample includes a convenience sample of six of the fifteen current ACGME pediatric subspecialties offering training programs. While we chose these subspecialties because of their potential contributions to global health, and that together they account for the majority of ACGME pediatric subspecialty training programs and fellows, the data from this sample may not represent the availability or type of global health opportunities within all other pediatric subspecialty training programs. Furthermore, though we recognize and applaud institutions that offer dedicated pediatric global health fellowships [16-18], these training programs are not currently ACGME-accredited and hence our assessment did not include the availability of global health opportunities therein. We would, however, expect that global health opportunities are more extensive in these fellowship programs than those within most ACGME-accredited subspecialty fellowship training programs.

## Conclusions

With the global shortage of healthcare workers and children worldwide suffering illness and death, many US pediatric residents are interested in working abroad, of whom even more are likely to subspecialize. Our results reveal that among a sample of ACGME-accredited pediatric subspecialty fellowship training programs, there is limited but increasing report of global health opportunities and the types of these opportunities differ among subspecialties. Further research on this topic will be helpful in assessing the continued growth of opportunities in pediatric subspecialty fellowship training programs, and whether such opportunities in training translate to improved global child health.

## Abbreviations

ACGME: Accreditation Council for Graduate Medical Education; AM: Adolescent medicine; AMA-FREIDA: American Medical Association-Fellowship and Residency Electronic Interactive Database Access; CC: Critical care; EM: Emergency medicine; H/O: Hematology/Oncology; ID: Infectious disease; NEO: Neonatology-perinatology; US: United States.

## Competing interests

The authors deny any competing interests. No funding or payment was given to anyone to do this research or produce the manuscript.

## Authors’ contributions

CD conceived the study. CD, JC and HC contributed to its design. All authors carried out data collection. CD performed initial analysis and drafted the manuscript. All authors read, edited and approved the final manuscript.

## Pre-publication history

The pre-publication history for this paper can be accessed here:

http://www.biomedcentral.com/1472-6920/13/88/prepub

## Supplementary Material

Additional file 1: Table S1 Classification of global health opportunities by data source. Global health opportunities in pediatric subspecialty fellowship Additional file 1.docx. Click here for file

Additional file 2: Table S2 Global health opportunities by pediatric subspecialty and data source: 2008 and 2011. Global health opportunities in pediatric subspecialty fellowship Additional file 2.docx. Click here for file

Additional file 3: Table S3 Types of global health opportunities on individual program websites: 2008 and 2011. Global health opportunities in pediatric subspecialty fellowship Additional file 3.docx. Click here for file

## References

[B1] World Health Organization Children: reducing mortalityhttp://www.who.int/mediacentre/factsheets/fs178/en/index.html

[B2] United Nations Millennium Development Goals Report 2010 http://www.un.org/millenniumgoals/pdf/MDG%20Report%202010%20En%20r15%20-low%20res%2020100615%20-.pdf#page=28

[B3] World Health Organization The global burden of disease: 2004 updatehttp://www.who.int/healthinfo/global_burden_disease/2004_report_update/en/index.html

[B4] World Health Organization The world health report 2006: working together for healthhttp://www.who.int/whr/2006/whr06_en.pdf

[B5] Association of American Medical Colleges 2012 Medical School Graduation Questionnairehttps://www.aamc.org/download/300448/data/2012gqallschoolssummaryreport.pdf

[B6] Anspacher M Frintner MP Denno D Pak-Gorstein S Olness K Spector J O’Callahan C Global health education for pediatric residents: a national survey Pediatrics 2011 128 4 e959 e965 10.1542/peds.2011-0129 21911354

[B7] Castillo J Castillo H DeWitt TG Opportunities in global health education: a survey of the virtual landscape J Grad Med Educ 2011 3 3 429 432 22942980 10.4300/JGME-03-03-31 PMC3179205

[B8] Nelson BD Saltzman A Lee PT Bridging the global health training gap: Design and evaluation of a new clinical global health course at Harvard Medical School Med Teach 2012 34 1 45 51 10.3109/0142159X.2011.577122 21592020

[B9] Redwood-Campbell L Pakes B Rouleau K MacDonald CJ Arya N Purkey E Schultz K Dhatt R Wilson B Hadi A Pottie K Developing a curriculum framework for global health in family medicine: emerging principles, competencies, and educational approaches BMC Med Educ 2011 11 46 10.1186/1472-6920-11-46 21781319 PMC3163624

[B10] Howard CR Gladding SP Kiguli S Andrews JS John CC Development of a competency-based curriculum in global child health Acad Med 2011 86 4 521 528 10.1097/ACM.0b013e31820df4c1 21346499

[B11] Garfunkel LC Howard CR Expand education in global health: it is time Acad Pediat 2011 11 4 260 262 10.1016/j.acap.2011.06.001 21764014

[B12] Accreditation Council of Graduate Medical Education Data Resource Book, Academic Year 2011–2012 http://www.acgme.org/acgmeweb/Portals/0/PFAssets/PublicationsBooks/2011-2012_ACGME_DATABOOK_DOCUMENT.pdf23289500

[B13] Embi PJ Desai S Cooney TG Use and utility of Web-based residency program information: a survey of residency applicants J Med Internet Res 2003 5 3 e22 10.2196/jmir.5.3.e22 14517113 PMC1550569

[B14] Eneriz-Wiemer M Nelson BD Bruce J Chamberlain LJ Global health training in pediatric residency: a qualitative analysis of faculty director insights Acad Pediatr 2012 12 3 238 244 10.1016/j.acap.2012.02.005 22503444

[B15] Suchdev PS Shah A Derby KS Hall L Schubert C Pak-Gorstein S Howard C Wagner S Anspacher M Staton D O’Callahan C Herran M Arnold L Stewart CC Kamat D Batra M Gutman J A proposed model curriculum in global child health for pediatric residents Acad Pediatr 2012 12 3 229 237 10.1016/j.acap.2012.02.003 22484282

[B16] Nelson BD Herlihy JM Burke TF Proposal for fellowship training in pediatric global health Pediatrics 2008 121 6 1261 1262 10.1542/peds.2008-0932 18519498

[B17] Mandalakas AM Chandawarkar A Holsinger E Global child health fellowship programs in demand Pediatrics 2008 122 6 1417 1418 author reply 1418 19047273 10.1542/peds.2008-2313

[B18] Nelson BD Izadnegahdar R Hall L Lee PT Global health fellowships: a national, cross-disciplinary survey of US training opportunities J Grad Med Educ 2012 4 2 184 189 23730439 10.4300/JGME-D-11-00214.1 PMC3399610

