# Supplementary material for: Global health opportunities within pediatric subspecialty fellowship training programs: surveying the virtual landscape
Source: BMC Med Educ. 2013 Jun 20;13:88. doi: 10.1186/1472-6920-13-88 (PMC3691626; doi:10.1186/1472-6920-13-88)
Supplement: Additional file 1: Table S1 — Classification of global health opportunities by data source. Global health opportunities in pediatric subspecialty fellowship Additional file 1.docx. [file 1472-6920-13-88-S1.docx]

| **Table 1** **Classification of global health opportunities by data source** | |
| --- | --- |
| **AMA-FREIDA** | **Individual program websites (with examples)** |
| Yes - international experience | Yes - international opportunities |
| No - international experience | Elective - "Fellows interested in global health can arrange a one-month elective in the country of their choice." |
| No information posted | Research - “Research opportunities are available in the developing world.” |
|  | Track - "In addition to the standard curriculum … additional training and experience in global health." |
|  | "Other" - "Global medicine with optional field trips" and "MPH in global health" |
|  | No information posted |
