# Supplementary material for: Global health opportunities within pediatric subspecialty fellowship training programs: surveying the virtual landscape
Source: BMC Med Educ. 2013 Jun 20;13:88. doi: 10.1186/1472-6920-13-88 (PMC3691626; doi:10.1186/1472-6920-13-88)
Supplement: Additional file 2: Table S2 — Global health opportunities by pediatric subspecialty and data source: 2008 and 2011. Global health opportunities in pediatric subspecialty fellowship Additional file 2.docx. [file 1472-6920-13-88-S2.docx]

| **Table 2 Global health opportunities by pediatric subspecialty and data source: 2008 and 2011** | | | | | | | | | | | | | | | | |  |  |  | |  |  |  | |  |  |  |
| --- | --- | --- | --- | --- | --- | --- | --- | --- | --- | --- | --- | --- | --- | --- | --- | --- | --- | --- | --- | --- | --- | --- | --- | --- | --- | --- | --- |
|  | **Adolescent**  **Medicine** | | |  | | **Critical Care** | |  | **Emergency**  **Medicine** | |  | **Hematology / Oncology** | |  | **Infectious Disease** | | |  | **Neonatology - Perinatology** | | |  | **TOTAL** | | |  |  |
|  | 2008 | 2011 |  | | 2008 | | 2011 |  | 2008 | 2011 |  | 2008 | 2011 |  | 2008 | 2011 | |  | 2008 | 2011 | |  | 2008 | 2011 | |  |  |
|  | (N=25) | (N=26) | *P* | | (N=60) | | (N=63) | *P* | (N=46) | (N=49) | *P* | (N=66) | (N=66) | *P* | (N=61) | (N=60) | | *P* | (N=97) | (N=96) | | *P* | (N=355) | (N=360) | | *P* |  |
| **AMA-FREIDA**  **N (%)** |  |  |  | |  | |  |  |  |  |  |  |  |  |  |  | |  |  |  | |  |  |  | |  |  |
| Yes | 1 (4) | 3 (12) | 0.61 | | 10 (17) | | 21 (33) | 0.04 | 12 (26) | 15 (31) | 0.66 | 7 (11) | 12 (18) | 0.32 | 14 (23) | 15 (25) | | 0.83 | 13 (13) | 16 (17) | | 0.55 | 57 (16) | 82 (23) | | 0.02 |  |
| No | 4 (16) | 8 (31) |  | | 25 (42) | | 19 (30) |  | 8 (17) | 9 (18) |  | 32 (49) | 30 (46) |  | 9 (15) | 11 (18) | |  | 47 (49) | 42 (44) | |  | 125 (35) | 119 (33) | |  |  |
| No information | 20 (80) | 15 (58) |  | | 25 (42) | | 23 (37) |  | 26 (57) | 25 (51) |  | 27 (41) | 24 (36) |  | 38 (62) | 34 (57) | |  | 37 (38) | 38 (40) | |  | 173 (49) | 159 (44) | |  |  |
|  |  |  |  | |  | |  |  |  |  |  |  |  |  |  |  | |  |  |  | |  |  |  | |  |  |
| **Program websites**  **N (%)** | |  |  | |  | |  |  |  |  |  |  |  |  |  |  | |  |  |  | |  |  |  | |  |  |
| Yes | 1 (4) | 1 (4) | 1.00 | | 0 (0) | | 5 (8) | 0.06 | 6 (13) | 10 (20) | 0.42 | 2 (3) | 8 (12) | 0.10 | 15 (24) | 20 (33) | | 0.32 | 6 (6) | 12 (13) | | 0.15 | 30 (8) | 56 (16) | | 0.004 |  |
| No information | 24 (96) | 25 (96) |  | | 60 (100) | | 58 (92) |  | 40 (87) | 39 (80) |  | 64 (97) | 58 (88) |  | 47 (76) | 40 (67) | |  | 91 (94) | 84 (88) | |  | 326 (92) | 304 (84) | |  |  |

Percentages may not add up to 100 due to rounding.
