# Supplementary material for: Global health opportunities within pediatric subspecialty fellowship training programs: surveying the virtual landscape
Source: BMC Med Educ. 2013 Jun 20;13:88. doi: 10.1186/1472-6920-13-88 (PMC3691626; doi:10.1186/1472-6920-13-88)
Supplement: Additional file 3: Table S3 — Types of global health opportunities on individual program websites: 2008 and 2011. Global health opportunities in pediatric subspecialty fellowship Additional file 3.docx. [file 1472-6920-13-88-S3.docx]

| **Table 3 Types of global health opportunities on individual program websites: 2008 and 2011** | | | | | | | |  |
| --- | --- | --- | --- | --- | --- | --- | --- | --- |
|  | **Adolescent Medicine** | **Critical Care** | **Emergency Medicine** | **Hematology / Oncology** | **Infectious Cisease** | **Neonatology-Perinatology** | **TOTAL** |  |
|  | 2008 2011  (N=1*) (N=1) | 2008 2011  (N=0) (N=5) | 2008 2011  (N=6*) (N=10*) | 2008 2011  (N=2*) (N=8*) | 2008 2011  (N=15*) (N=20*) | 2008 2011  (N=6) (N=12*) | 2008 2011  (N=30*) (N=56*) |  |
| Electives | 0 1 | 0 1 | 4 4 | 1 4 | 4 5 | 0 3 | 9 18 |  |
| Research | 1 0 | 0 4 | 2 3 | 1 5 | 5 11 | 2 5 | 11 28 |  |
| Track | 1 0 | 0 0 | 1 2 | 0 0 | 0 0 | 0 0 | 2 2 |  |
| Other | 0 0 | 0 0 | 1 3 | 1 0 | 8 10 | 4 6 | 14 19 |  |
| TOTAL | 2 1 | 0 5 | 8 12 | 3 9 | 17 26 | 6 14 | 36 67 |  |

*Some programs with more than one type of opportunity listed on their individual website.
